# Influenza Virus—Host Co-evolution. A Predator-Prey Relationship?

**DOI:** 10.3389/fimmu.2018.02017

**Published:** 2018-09-07

**Authors:** Konstantinos Voskarides, Eirini Christaki, Georgios K. Nikolopoulos

**Affiliations:** Medical School, University of Cyprus, Nicosia, Cyprus

**Keywords:** adaptation, antagonistic evolution, influenza virus, bottleneck, immune system, antigen, genetics, mutation

## Abstract

Influenza virus continues to cause yearly seasonal epidemics worldwide and periodically pandemics. Although influenza virus infection and its epidemiology have been extensively studied, a new pandemic is likely. One of the reasons influenza virus causes epidemics is its ability to constantly antigenically transform through genetic diversification. However, host immune defense mechanisms also have the potential to evolve during short or longer periods of evolutionary time. In this mini-review, we describe the evolutionary procedures related with influenza viruses and their hosts, under the prism of a predator-prey relationship.

## Introduction

Health disasters caused by influenza viruses are abundant in human history (1–3). The Spanish flu of 1918 is estimated to have claimed more than 50 million lives, a figure that was beyond the death toll of World War I (4, 5). Another two pandemics occurred later in the twentieth century when surveillance systems and laboratory capacity were in place to monitor them: the Asian flu (1957) and the Hong Kong flu (1968) (2, 6), which had lower fatality rates but still caused between half and 2 million deaths each. In 2009, a novel H1N1 influenza virus spread rapidly from Mexico to the rest of the globe (2) but had eventually moderate impact with less deaths than the previous pandemics (2, 7), partly due to our improved response. Seasonal epidemics may also be severe with high morbidity and mortality (8).

Viral infections are not a static phenomenon. Many viral genomes can adapt on their hosts' cellular environment while survivors of viral infections have probably special genetic characteristics that helped them remain alive. Therefore, both genomes change over time reminding us of the evolutionary race between predators and preys. Viral genomes however are smaller and more flexible, allowing viruses to rapidly adapt though random mutagenesis or due to transmission in other species. Pandemics of influenza are due to these random and sudden viral mutations or trans-species transmissions. Under this perspective, in this paper, we discuss about influenza viruses and the coevolution with the human genomes.

## Influenza virus

### Virology and replication cycle

Influenza viruses are members of the *Orthomyxoviridae* family. Their genome is comprised of a negative sense, segmented single-stranded RNA. Viral particles also contain essential viral proteins and a host cell-derived envelope (9). Influenza viruses are further classified into four types: A, B, C, and D. Influenza virus types A, B, and C infect and cause respiratory illness in humans. Influenza D viruses mainly affect cattle and are not known to infect humans (10). Influenza A and B viruses cause seasonal epidemics whereas type C viruses usually cause a mild upper respiratory tract illness and associated epidemics have only been scarcely reported (11). Influenza A viruses can infect many animal species, including birds, pigs, horses, marine mammals, and other hosts, and can cause pandemics. Influenza A viruses are categorized into subtypes based on the molecular characteristics of their surface glycoproteins, hemagglutinin (HA), and neuraminidase (NA). Identification of at least 18 antigenically distinct HA subtypes and 11 distinct NA subtypes of influenza A virus strains infecting humans and animals have so far been determined (12, 13). Two genetically and antigenically distinct lineages (Victoria and Yamagata) of Influenza B viruses co-circulate in humans (14–16).

Hemagglutinin is comprised of a dimer HA1-HA2: HA1 is crucial for binding to the host cell receptor whereas HA2 for cell fusion. Viral endocytosis is followed by uncoating and release of viral RNA, which is imported into the nucleus where viral replication and protein synthesis take place using viral polymerase proteins and the host cell machinery (17). Virions are assembled in the cell surface and bud enclosed in an envelope originating from the host cell membrane. Neuraminidase allows the virus to leave the infected cell as it cleaves sialic acid (SA) from the cell surface receptors. Viral replication causes cell death with various mechanisms including disruption of protein synthesis and apoptosis. Since viral release continues for hours before cell death, many respiratory epithelial cells are affected and die within a few replication cycles (9, 18).

Influenza viruses target epithelial cells of the respiratory tract, which contain SA receptors. Epithelial cells across species express different SA receptors and Influenza A virus strains show a predilection for certain types of such receptors, making zoonotic transmission difficult. For example, human influenza strains have a predilection for SA α-2,6 galactose receptors, which are found in the respiratory epithelium of the upper airways in humans, while animal influenza A viruses bind to SA α-2,3 galactose, which is found on the epithelial cells of birds and pigs, but could also be expressed in the human lower respiratory tract epithelium (12, 19, 20). HA epitopes are the major determinants for the production of strain-specific neutralizing antibodies.

### Clinical manifestations

Influenza symptoms usually present abruptly, after an incubation period of 1–2 days. Systemic symptoms are characteristic and help differentiate influenza from other upper respiratory tract viral illnesses. These include high fever, chills, rigors, headache, myalgias, malaise, and anorexia. Fever and systemic symptoms commonly last for 3 days, however fever can last up to 8 days. Myalgias can be severe and usually involve the back and extremities. Respiratory symptoms include dry cough, sore throat, hoarseness, nasal congestion, and discharge (18).

Different subtypes of influenza have different ability to infect airway epithelial cells of the upper or lower respiratory tract, hence causing a milder infection or a more severe illness leading to severe pneumonia. For example, H5N1 infects alveolar epithelial cells as well as alveolar macrophages, triggering a significant pro-inflammatory response, which can result in severe lung injury (21–23).

## Host immune response to influenza virus infection

Cells of the innate immune response are the first and fast responders upon influenza virus infection, recruited by chemokines released by airway epithelial cells. Upon viral entry, intracellular viral ssRNA and other viral molecular patterns are recognized mainly by Toll-like receptors (TLR) 3,7,8,9 and retinoic acid-inducible gene-I protein (RIG-1) receptors. The downstream signaling triggered by the activation of these receptors results in the activation of transcription factors like nuclear factor kappa-B and interferon regulatory factor (IRF) 3 and 7, leading to the expression of pro-inflammatory cytokines and interferons (24–26). Moreover, NOD-like receptor family pyrin domain containing 3 (NALP3) inflammasome is also activated upon influenza virus infection promoting IL-1β and IL-18 secretion, and pulmonary infiltration by neutrophils and macrophages (27).

Natural Killer (NK) cells, monocytes, neutrophils, and dendritic cells migrate to the site of infection and exhibit antiviral activity. NK cells have cytotoxic activity on cells infected with influenza virus, macrophages phagocytose infected cells and regulate adaptive immune responses, and dendritic cells present viral antigens bound to Major Histocompatibility Complex (MHC) molecules to naïve and memory T lymphocytes, initiating the specific adaptive immune response. In addition, immunoglobulins (mainly IgA) present in nasal secretions contribute to the anti-influenza immune response by preventing viral entry (26).

Both T and B cells are essential in the adaptive immune response to influenza virus infection. Naive CD8^+^ T cells, upon activation by dendritic cells and facilitated by the action of cytokines, proliferate and differentiate to cytotoxic T lymphocytes (CTLs). CTLs are able to kill influenza virus-infected cells and also restrict viral replication via production of cytokines and effector molecules like perforin and granzymes (26, 28, 29). Memory CTLs cells can respond efficiently during a secondary infection and confer cross-protective heterosubtypic immunity (30, 31). Moreover, CD4^+^ T cells express co-stimulatory molecules that participate in antibody production by B cells. At the same time, antiviral cytokines, like INF-γ, TNF, and IL-2 are expressed from Th1 CD4^+^ T cells, which also help activate alveolar macrophages. Th2 CD4^+^ T cells contribute to B cell specific responses (32). Comprising an essential defense mechanism against influenza virus infection, B cells generate neutralizing (hemagglutinin-inhibiting antibodies) as well as non-neutralizing antibodies (anti-NA and antibodies against structural proteins). The latter, as well as specific antibody dependent cell-mediated cytotoxicity, play an important role in cross-protection against different influenza subtypes (31, 33). There is increased interest in the role of cross-reactive T cells and broadly neutralizing antibodies in the development of vaccines that could elicit “universal” immunity against different or novel strains of influenza viruses.

## Viral evolution

### Predator and prey. antagonistic evolution

Predator-prey species relationship is of great interest in evolutionary biology since the balance of attack and defense mechanisms is very important for the long-term survival of both species. A simplified example that is often given is that of lions and their prey. The faster the lions are, the most successful they can be in acquiring food, surviving, and reproducing. The faster the zebras (lions' prey) are, the better they can escape the lions, so they survive and reproduce. It is all about Darwinian fitness, since survival and robustness are related with a higher probability of having more offspring (descendants). That is translated to reproducibility and to a better chance to evolve. Darwinian fitness cannot increase simultaneously for both predator and prey species. It increases for one species and reduces for the other. But that is not stable. Afterwards the opposite happens. This is the phenomenon of antagonistic evolution that is believed to push evolution forward and sometimes can increase phenotypic diversification (34).

Antagonistic evolution can be a main driver of multiple phenotypes, specialization, or even speciation. Multiple species evolution, those belonging to the same genus or family, is termed as evolutionary radiation. An example is that of marine mammals, dolphins, and whales. It is believed that multiple such species have been evolved due to prey (fish, squid) specialization (35, 36).

Antagonistic evolution also happens between host and various pathogens including viruses. Successful viruses are the ones that can cheat cells, go inside the cytoplasm, take advantage of the cell translation system (ribosomes), reproduce, and then exit the cell by exocytosis going to infect other cells. Viruses can be considered as the predators and cells as the preys, even though viruses do not necessarily kill the cells. This is probably a major difference with the classical predator-prey relationship. Evolutionary successful viruses are transmitted from one host to another (cell or a whole organism), making minimal damage. On the other site, cellular defense mechanisms, like the MHC proteins, have the potential for evolving in a quite short evolutionary time, this being a highly effective immunological shield.

### Genetic diversification of influenza viruses

Influenza viruses exhibit vast genetic variability sourced from three molecular mechanisms: mutation, genetic re-assortment, and genetic recombination. While the first two mechanisms have been extensively studied, the significance of the third one needs clarification (37). The results of these genetic procedures are antigenic shift or antigenic drift. Antigenic shift is a rapid change of the virus genome and antigenicity, coming from a combination of different viral strains. The eight RNA fragments of the influenza virus can re-assort between different strains, producing new subtypes. This happens usually inside livestock animals, in geographic regions where people live in proximity with animals (38). Antigenic shifts caused influenza pandemics in the past. On the other hand, mutations, usually nucleotide substitutions, can cause antigenic drifts. Antigenic drifts cause more gradual changes to the viral HA and N proteins, this being the cause of the appearance of new influenza strains each year (39). Antigenic drifts are very important for vaccine production.

The major cause of the high evolutionary rate of influenza viruses is the lack of proofreading (correction) function of the viral RNA polymerase. Mutagenesis is close to 10^−3^-10^−4^ per nucleotide site, this being a very high mutagenesis rate and producing a high genetic variability in the viral genetic pool (40, 41). Another consequence of the continuously evolved viral genetic variability is the increased probability for widening the host-range of the virus due to HA protein changes (37).

Influenza A probably evolves under a negative pressure for CpG oligonucleotides. This is considered to happen due to innate immune recognition, something that is not so vital for influenza B evolution (42, 43). Additionally, it has been shown that antigenic drifts are more frequent to influenza A viruses than influenza B viruses (44). Even though antigenic drifts are usually gradual changes regarding the viral properties, the possibility for a rapid evolutionary change due to a mutation (punctuated evolution) cannot be excluded.

One of the most interesting recent findings regarding influenza virus' genetic variability and evolution is that when people become infected, they are actually infected from a diversity of viruses and not from only one strain (45). This implies that vaccines target the dominant strain and not the whole population of viruses. Most of these viral strains are not well adapted but can stay for long inside a human (or other species) population, thus hiding the danger to gain a gradual adaptation through mutation accumulation processes (46–48). It is obvious that natural selection can determine which viral variants can survive and which cannot. Additionally, this multi-infection process may be related with cooperation or competition phenomena among viruses, that currently are not so well understood (39).

Further understanding of the mutational and evolutionary procedures related to influenza viruses can help us develop prediction models. Prediction models are very important for vaccine production. Most of the predictive modeling studies focus on the surface protein hemagglutinin (49). Hemagglutinin DNA sequences for thousands of influenza A strains, isolated over the last 40 years, are freely available in electronic databases (50). However, prediction studies and experimental evolution studies are very challenging. Currently, available tools are not adequate for a reliable approach. More details can be found in the recently published reviews (51) and (52).

### A predator—prey relationship between human and influenza virus

The relationship between viruses and eukaryotic cells has its origins millions of years ago. It is not so simple since many viruses complete their cycle inside a cell without harming it. Instead, sometimes their nucleic acid sequences have been embedded in the host cell DNA. In humans, about half of their genome is constituted of viral sequences, showing this long lasting ancient interaction (53, 54).

**Figure 1:**
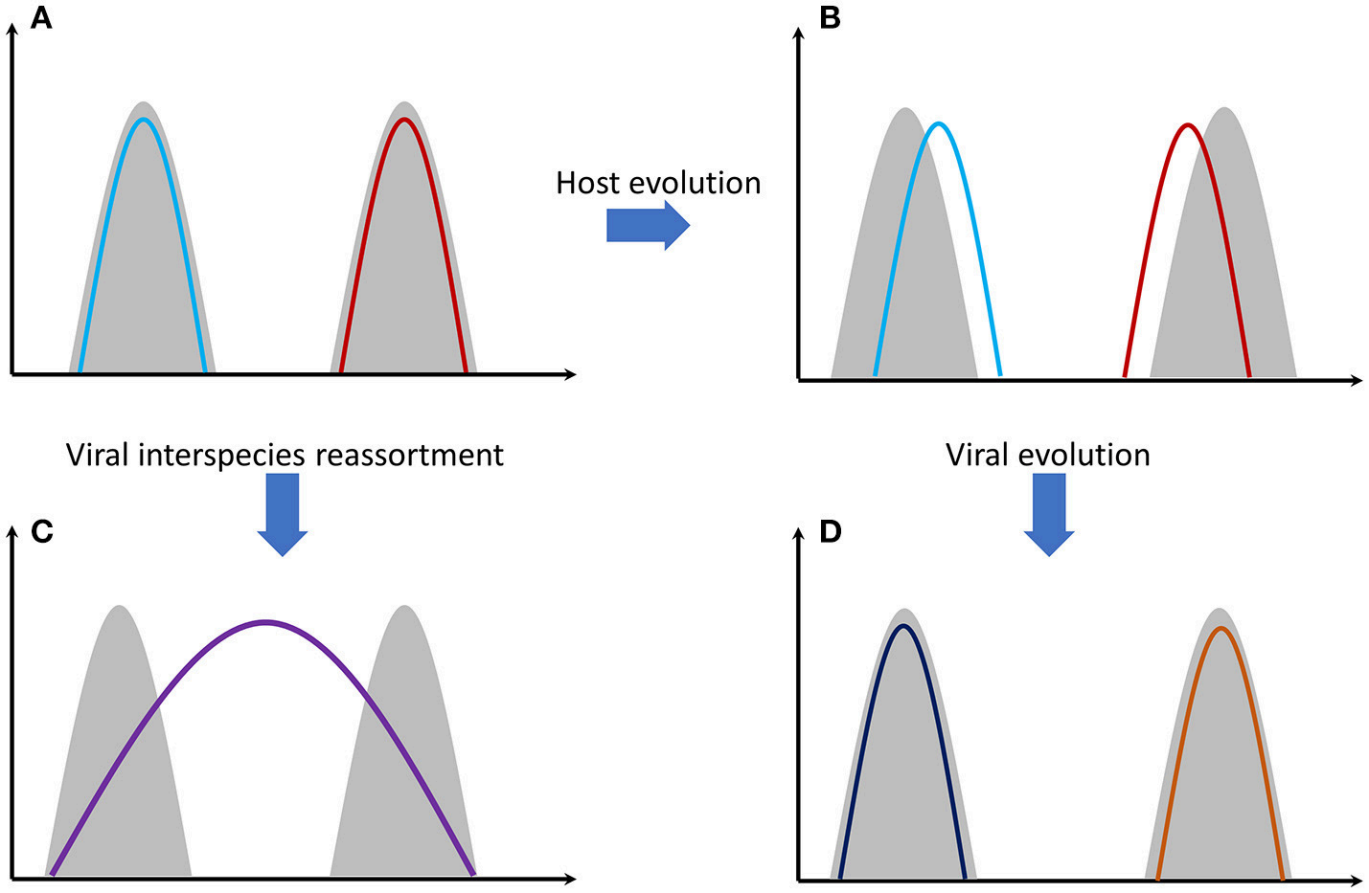
Gray shading represents host genomes of two different species. Colored lines represent the viral genomes. **(A)** Genomes of two different viral strains, e.g., influenza, that are perfectly adapted to their hosts' cells. **(B)** Host genomes have evolved through mutations and natural selection. Viral adaptation has been reduced. **(C)** The two viral strains have been genetically re-assorted since they have met in the same individual(s). The new recombined strain can potentially infect both species. Mortality can be high since the new viral strain needs time to adapt effectively to its hosts' cells. **(D)** Viral strains evolved again through random mutations and natural selection. Viral adaptation has been increased again.

Antibody-mediated immunity to influenza viruses is well-understood. Vaccines success is based on this response. Despite this, vaccines are ineffective in many people. One reason is that cellular immunity through T cells is also a very important immunological response (32). Antibody variability is a genetically intrinsic mechanism of B cells. However, T-cell response is based on MHC (HLA) proteins efficiency that differs among individuals and is an inherited trait. Recognition of viral proteins on the surface of infected cells is performed through MHC proteins. There are thousands of genetic polymorphisms in the MHC genes at the population level, producing millions of combinations. These combinations are inherited in the form of “genes packages” termed as haplotypes (each person carries two MHC haplotypes). It is unlikely for two individuals to carry a single common haplotype. Some MHC proteins (haplotypes) are more effective than others in recognizing viral proteins. Why is there this vast diversity of MHC proteins in human populations? The answer is that this increases the likelihood that some individuals will survive after a severe pandemic and this way a species extinction is avoided. On the other hand, we can assume that haplotypes that exist today are the ones that were successful in the past for combating specific viral or bacterial infections. Survivors passed these haplotypes onto next generations. Of course, as it was stated before, viruses are not stable entities. Mutations increase the number of viral types and some of them will finally “win” the previously successful MHC proteins causing again the (human) population to be susceptible. This is quite similar with the predator-prey relationship happening extensively in nature (Figure 1).

T4 cells (CD4 cells or T-helper cells) and T8 cells (CD8 cells or T-cytotoxic cells) are the most important lymphocytes that use MHC proteins for recognizing the virus-infected cells. Studies showed that the long-term evolution of T8 cells epitopes may have shaped the long-term evolution of influenza A virus (55, 56). On the other hand, influenza viruses seem to respond to this evolutionary pressure by mutations to their nucleoprotein gene (nucleoprotein is an important viral capsid protein for T-cell recognition). It has also been proven that some combinations of MHC proteins and nucleoprotein variants result in immunological response and some not (57–59). This antagonistic evolutionary relationship is a factor of a vice-versa increasing genetic variability. In the same regard, TLRs may have remained evolutionary stable, possibly due to the fact that the pathogen-associated molecular patterns (PAMPs) they recognize have also remained unaltered, since they are fundamental for pathogen survival (60).

Another category of important proteins in human cells, defending against viral infections, are the IFITM (interferon-induced transmembrane) proteins. At least three human IFITM proteins exist (61). When upregulated (by both type I and type II interferons), they can restrict viruses outside cells, prohibiting viral penetration through the membrane lipid layers. IFITM3 seems to be more specific for influenza viruses (61). Gene polymorphisms in IFITM3 gene can increase or reduce susceptibility to influenza infections, underlining the evolutionary significance of these proteins (62, 63).

### Genetic bottlenecks

In population genetics, genetic bottleneck is the event where a sub-group of individuals originating from a larger population, migrate in another geographic region, carrying only a subset of the genetic information of the initial population. This is a standard procedure during evolution. Populations split, and a part of the initial genetic diversity is transferred in different geographic regions. Isolation and new mutations can dramatically change the genetic diversity of the new population.

Transmission of viruses in new hosts implies three different genetic bottleneck phenomena: (i) The viral transmission bottleneck, which determines how much of the viral diversity generated in one host passes to another during transmission (64, 65). Studies show that along with major variants, other minor viral variants can be transferred from one host to another (66). These minor variants can be transformed to major through natural selection pressures. Two different processes are related with this bottleneck: virus entry and virus replication followed by production of new virions (ii) Individuals or sub-populations (e.g., humans) migrate in other geographic regions and transfer there only a group of viruses of the initial pool of viruses, and (iii) Genetic bottlenecks of the hosts, e.g., humans. Host genome is vital for the effectiveness of the immune response. Only a subset of the paternal population MHC genes' variability will be transferred in the new geographic region.

**Figure 2:**
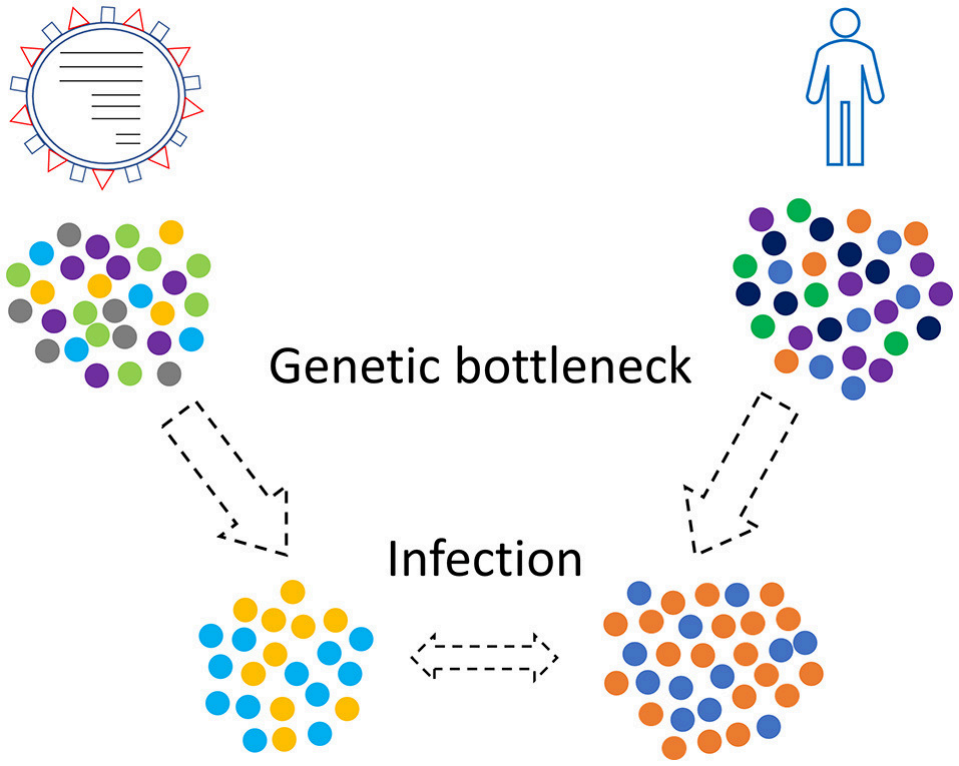
Genetic bottleneck phenomena occur in viruses and humans as well. Only a subset of the initial populations, viruses and humans, will finally come in contact.

Combinations of those three kinds of genetic bottlenecks and evolutionary forces will shape the final virus-host relationship that will take place in a human population (Figure 2). This relationship is not static since new genetic bottlenecks take place, especially in present days that people travel a lot.

What determines the viral bottleneck size? Small viral founding populations are usually observed, even though this depends on the virus and the host (67). Experimental studies in ferret and guinea pig showed that influenza infections coming from limited dose of aerosol exposure had tighter bottlenecks than contact transmission (68). More studies are needed to elucidate the importance of this phenomenon in human populations. Viruses follow special routes in our planet and understanding this may contribute to prevention strategies.

## Conclusion

Influenza viruses and humans have co-evolved in the past and will continue to co-evolve. Our understanding of influenza virus pathogenesis and evolution has increased, however, we are still unprepared to stop the next pandemic strain from emerging. Evolution prediction algorithms can help inform efforts toward the development of better preventive measures.

## Author contributions

KV and EC: conception, drafting the manuscript, approval of the final version; GN: conception and design, critical revision of the manuscript, approval of the final version.

### Conflict of interest statement

The authors declare that the research was conducted in the absence of any commercial or financial relationships that could be construed as a potential conflict of interest.
